# Ipilimumab and radiation therapy for melanoma brain metastases

**DOI:** 10.1002/cam4.140

**Published:** 2013-10-10

**Authors:** Ann W Silk, Michael F Bassetti, Brady T West, Christina I Tsien, Christopher D Lao

**Affiliations:** 1 Division of Hematology/Oncology, Department of Internal Medicine, University of Michigan Comprehensive Cancer CenterAnn Arbor, Michigan; 2 Department of Human Oncology, University of Wisconsin Carbone Cancer CenterMadison, Wisconsin; 3 Institute for Social Research, University of MichiganAnn Arbor, Michigan; 4 Department of Radiation Oncology, University of MichiganAnn Arbor, Michigan

**Keywords:** Brain metastases, immunotherapy, ipilimumab, melanoma, stereotactic radiosurgery

## Abstract

Ipilimumab, an antibody that enhances T-cell activation, may augment immunogenicity of tumor cells that are injured by radiation therapy. We hypothesized that patients with melanoma brain metastasis treated with both ipilimumab and radiotherapy would have improved overall survival, and that the sequence of treatments may affect disease control in the brain. We analyzed the clinical and radiographic records of melanoma patients with brain metastases who were treated with whole brain radiation therapy or stereotactic radiosurgery between 2005 and 2012. The hazard ratios for survival were estimated to assess outcomes as a function of ipilimumab use and radiation type. Seventy patients were identified, 33 of whom received ipilimumab and 37 who did not. The patients who received ipilimumab had a censored median survival of 18.3 months (95% confidence interval 8.1–25.5), compared with 5.3 months (95% confidence interval 4.0–7.6) for patients who did not receive ipilimumab. Ipilimumab and stereotactic radiosurgery were each significant predictors of improved overall survival (hazard ratio = 0.43 and 0.45, with *P* = 0.005 and 0.008, respectively). Four of 10 evaluable patients (40.0%) who received ipilimumab prior to radiotherapy demonstrated a partial response to radiotherapy, compared with two of 22 evaluable patients (9.1%) who did not receive ipilimumab. Ipilimumab is associated with a significantly reduced risk of death in patients with melanoma brain metastases who underwent radiotherapy, and this finding supports the need for multimodality therapy to optimize patient outcomes. Prospective studies are needed and are underway.

## Introduction

Brain metastases from melanoma are prevalent and clinically devastating. Most patients who develop melanoma brain metastases (MBM) die of neurologic sequelae [1]. Local therapy, such as surgery or radiation, has traditionally been the mainstay of treatment. The use of whole brain radiation therapy (WBRT) may impact on neurologic deaths of patients with MBM as compared to best supportive care, but the overall survival (OS) following WBRT remains dismal at 3–4 months [2]. Stereotactic radiosurgery (SRS) is often used for limited numbers of small metastases, but the median survival following SRS is only 5–6 months [3, 4].

Ipilimumab, an antibody that blocks the cytotoxic T-lymphocyte antigen-4 (CTLA-4) immune checkpoint, was approved by the United States Food and Drug Administration (FDA) based on an OS advantage in patients with metastatic melanoma [5]. Patients with active, untreated brain metastases were excluded from the phase III trials [5, 6]. In a dedicated phase II study of ipilimumab in patients with brain metastases, Margolin et al. [7] reported that ipilimumab had activity in the brain which was similar to systemic activity, with a response rate of 16% in neurologically asymptomatic subjects. Median survival was 3.7 months in patients who had neurologic symptoms at enrollment and 7.0 months in patients who did not. More than 40% of patients had previously received radiation to the brain prior to enrollment. In a retrospective study of 77 patients with MBM who underwent SRS, ipilimumab therapy was associated with a 16-month improvement in median survival over those that did not receive ipilimumab [8]. In a similar retrospective series of 58 patients, a 10% improvement in 6-month OS was seen, although it was not statistically significant [9]. Importantly, responses in the brain, as in extracranial disease, may be durable [10].

No data are currently available from trials of concurrent ipilimumab and radiation therapy (RT), although these are actively accruing. To better understand the effect of ipilimumab on the outcomes of patients with MBM and potentially guide the design of future clinical trials, we reviewed our experience with patients treated with WBRT and SRS who did and did not receive ipilimumab. Given the existing research in this area, we hypothesized that patients with melanoma brain metastasis treated with both ipilimumab and radiotherapy would have improved OS, and that the sequence of treatments may affect disease control in the brain.

## Methods

The clinical and radiographic records of patients with MBM at the University of Michigan between 2005 and 2012 were reviewed. Within that population, those patients who underwent RT for brain metastases and received one or more doses of ipilimumab (either before or after RT) were identified and included in the analysis. Ipilimumab was given intravenously at a dose of 3 mg/kg every 3 weeks for a planned four doses. A reinduction course was given for some patients who experienced disease control with the initial course. Whole brain radiotherapy and SRS were both included to explore the effects of radiation delivery, schedule, and dose intensity.

As a comparison group for the patients who received ipilimumab and WBRT, 21 patients with melanoma were identified from participation in a phase I clinical trial of WBRT with concurrent bortezomib as a potential radiosensitizer in 2007–2009 [11]. None of these participants received ipilimumab at any point during their course of treatment. As a comparison group for the patients who received ipilimumab and SRS, we identified serial cases from 2005 to 2011 who underwent SRS prior to the FDA approval of ipilimumab. This study was approved by the University of Michigan Institutional Review Board (IRB protocol #67184).

Outcomes of interest were OS, time to progression in the brain (TTP_br_), and proportion of patients with a response to RT. Data on the clinical courses of these patients were extracted from their medical records, including brain imaging, subsequent treatments, survival, and serum lactate dehydrogenase (LDH) level [12, 13]. Intratumoral hemorrhage [14] and radiation necrosis [15] were defined as present if noted explicitly as new or worsening signs of bleeding. We calculated prognostic scores by two commonly used methods: the recursive partitioning analysis (RPA) score [4] and the diagnosis-specific graded prognostic assessment (DS-GPA) for melanoma [16]. Both of these scales incorporate performance status and number of brain metastases, which are recognized as important prognostic variables. The distributions of baseline variables for patients either receiving or not receiving ipilimumab were compared using chi-square tests for categorical variables and two-sample *t*-tests for continuous variables. Survival was measured from the date of the first RT to the brain, and the events of interest were progression of brain metastases and death. Patients who had objective evidence of progression of brain metastases prior to death and who displayed altered mental status or failure to thrive leading up to the time of death were categorized as dying of brain metastases. Survival data were considered right censored at the date of last follow-up (2/15/13) if the events of interest had not been observed as of that date. Patients were evaluable for response if lesions were 5 mm or larger in longest diameter on baseline imaging and repeat imaging was performed 4–16 weeks after RT, prior to the patient receiving additional therapy. The proportion of patients with complete response (CR) or partial response (PR) to RT and TTP_br_ were classified using both response evaluation criteria in solid tumors (RECIST) [17] and immune-related response criteria (irRC) [18] as per Margolin et al. [7].

Descriptive statistics for the survival times and 95% confidence intervals for the median survival times were used to summarize the courses of our subjects. The nonparametric *K*-sample test was used to compare the equality of median survival times. Fisher's exact test was used to compare the proportions of responses between the ipilimumab and comparison groups. A Cox regression model was used to estimate the hazard ratios (HR) associated with the ipilimumab and SRS treatments, as both ipilimumab and fewer brain metastases have been associated with improved survival [4–6, 16]. In the Cox model, we also tested the significance of an interaction between the ipilimumab and SRS treatment indicators, and performed a post hoc power calculation for the interaction term (given our small sample sizes). Available commands in the Stata 12.1 software (*exact, median, stci, stcox,* and *sts graph*) were used for all analyses, and the R function *powerEpiInt()* in the R package *powerSurvEpi* was used for the post hoc power calculation.

## Results

Thirty-three patients with MBM received ipilimumab (12 before RT and 21 after RT). Among the patients who received ipilimumab, 16 underwent WBRT and 17 underwent SRS. The median number of doses of ipilimumab received was 4 (range, 1–8). Among the 37 patients in the comparison groups, 21 underwent WBRT (in a phase I trial of concurrent bortezomib) [11] and 16 underwent SRS. The average interval between the first dose of ipilimumab and RT was 23 weeks. There were no significant differences between the ipilimumab groups and the comparison groups with respect to age, sex, type of melanoma, number of brain metastases, prior craniotomy status, performance status, and prognostic indicators (Table 1). The frequency of patients who received any prior and subsequent systemic therapy was similar; however, 13 patients (39.4%) in the ipilimumab groups received BRAF inhibitor therapy, which was significantly more than in the comparison groups. Those patients received either vemurafenib or dabrafenib, which was available at the University of Michigan as part of a clinical trial. BRAF mutational status was known for all of patients in the ipilimumab groups, but only 11% in the comparison groups due to the year of treatment. Patients in the ipilimumab groups also received additional RT to the brain more frequently (54.6% vs. 8.1%).

**Table 1:** Characteristics of the study population

	No ipilimumab (*n*=37)	Ipilimumab (*n*=33)	*P*-value
Type of RT			
WBRT	21 (56.8%)	16 (48.5%)	0.49
SRS	16 (43.2%)	17 (51.5%)	
Years treated	2005–2011	2009–2012	Not applicable
Mean age (years)	57.7	56.6	0.76
Sex			
Female	17 (45.9%)	13 (39.4%)	0.58
Male	20 (54.1%)	20 (60.6%)	
Type of melanoma			
Cutaneous	31 (83.8%)	32 (97.0%)	0.13
Mucosal	2 (5.4%)	1 (3.0%)	
Unknown primary	4 (10.8%)	0 (0%)	
Number of brain metastases			
>3	16 (43.2%)	18 (54.6%)	0.39
2 or 3	9 (24.3%)	4 (12.1%)	
1	12 (32.4%)	11 (33.3%)	
Craniotomy prior to RT			
Yes	7 (18.9%)	6 (18.2%)	0.94
No	30 (81.1%)	27 (81.8%)	
ECOG PS			
0	16 (45.7%)	15 (53.6%)	0.15
1	12 (34.3%)	12 (42.9%)	
2–3^1^	7 (20.0%)	1 (3.6%)	
Neurologic symptoms			
Asymptomatic	20 (54.0%)	25 (75.8%)	0.06
Symptomatic	17 (46.0%)	8 (24.2%)	
RPA			
Class I	1 (2.7%)	0 (0%)	0.34
Class II	36 (97.3%)	33 (100%)	
DS-GPA			
0–1	8 (24.2%)	7 (25.0%)	0.99
2	12 (36.4%)	11 (39.3%)	
3	9 (27.3%)	7 (25.0%)	
4	4 (12.1%)	3 (10.7%)	
Serum LDH level			
Normal	20 (62.5%)	18 (64.3%)	0.89
Elevated	12 (37.5%)	10 (35.7%)	
BRAF status			
Mutated	3 (25.0%)	17 (51.5%)	0.37
Wild type	1 (75.0%)	16 (48.5%)	
Prior systemic therapy^2^			
Yes	19 (51.4%)	14 (42.4%)	0.46
No	18 (48.6%)	19 (57.6%)	
Subsequent systemic therapy^3^			
Yes	22 (62.9%)	18 (54.5%)	0.49
No	13 (37.1%)	15 (45.5%)	
Subsequent brain RT			
Yes	3 (8.1%)	18 (54.6%)	<0.001
No	33 (89.2%)	15 (45.5%)	
BRAF inhibitor ever			
Yes	1 (3.1%)	13 (39.4%)	<0.001
No	31 (96.9%)	20 (60.6%)	

RT, radiation therapy; WBRT, whole brain radiation therapy; SRS, stereotactic radiosurgery; PS, performance status; RPA, recursive partitioning analysis; DS-GPA, diagnosis-specific graded prognostic assessment; LDH, lactate dehydrogenase.

1 One patient in the ipilimumab group had an ECOG PS of 3 and the remainder had PS of 2.

2 Not including ipilimumab. Prior therapies included interferon, interleukin-2, tumor-infiltrating lymphocytes, cytotoxic chemotherapy, and BRAF inhibitors (four patients in the ipilimumab group).

3 Not including ipilimumab. Subsequent therapies included cytotoxic chemotherapy and BRAF inhibitors (nine patients in the ipilimumab group).

Thirty-seven patients received WBRT and 33 patients received SRS for the treatment of MBM. Patients who received WBRT were treated with 30–37.5 Gy in 10–13 fractions. The number of lesions present at the time of WBRT ranged from 1 to 62. Patients who were treated with SRS received 14–24 Gy in 1–5 fractions. The SRS treatment volume size ranged from 0.19 to 17.2 cc. The number of lesions present at the time of SRS ranged from 1 to 14.

**Figure 1:**
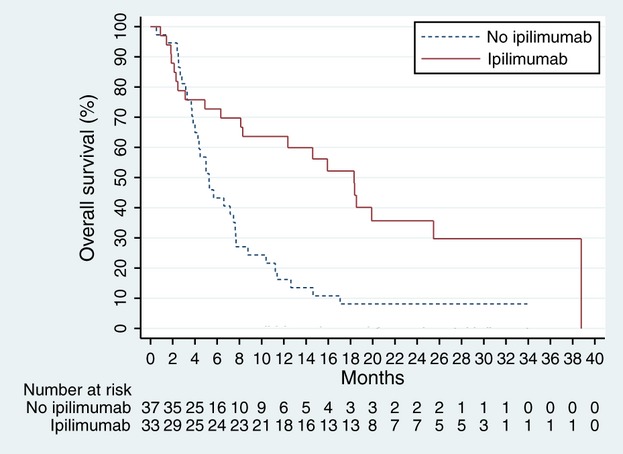
Censored overall survival of all patients by ipilimumab treatment. Treatment with ipilimumab was significantly associated with improved survival (HR = 0.43, *P* = 0.005). HR, hazard ratio.

Of the 70 patients, 55 died and the surviving 15 had at least 10 months of follow-up time. Patients who received ipilimumab had a median survival of 18.3 months, as compared with 5.3 months for those who did not receive ipilimumab (Table 2). In the Cox regression analysis, treatment with ipilimumab was found to be a statistically significant predictor of improved survival (HR = 0.43, *P* = 0.005; Fig. 1). Regarding the sequence of the therapies, the censored OS was 8.1 months for the patients who received ipilimumab before RT, versus 18.4 months for the patients who received ipilimumab after RT. Treatment with SRS as compared to WBRT was also a statistically significant predictor of improved survival (HR = 0.45, *P* = 0.008). Patients in the SRS groups had fewer brain metastases as compared to patients in the WBRT groups, with a median of 1 versus 6 brain metastases. Most patients died of their brain metastases (70% in the comparison groups and 81% in the ipilimumab groups). Median TTP_br_ using irRC was ∼3 months in all treatment groups (Table 2).

**Table 2:** Censored median TTP_br_ and OS (with 95% confidence interval) in months from date of first RT to brain

	No ipilimumab	Ipilimumab	*P*-value
TTP_br_			
All patients	3.3 (1.5–6.3)	2.7 (1.5–6.0)	0.55
WBRT	3.3 (1.4–6.3)	2.7 (1.0–8.2)	0.72
SRS	2.6 (not estimable^1^)	2.6 (1.2 to not estimable^2^)	0.95
OS			
All patients	5.3 (4.0–7.6)	18.3 (8.1–25.5)	0.002
WBRT	5.3 (4.3–7.6)	3.1 (1.9 to not estimable^2^)	0.60
SRS	4.0 (3.2–14.6)	19.9 (15.9 to not estimable^2^)	0.009

TTP_br_, time to progression in the brain; OS, overall survival; RT, radiation therapy; WBRT, whole brain radiation therapy; SRS, stereotactic radiosurgery.

1 The 95% confidence interval for the median survival time could not be determined because there were only five patients in this cell.

2 The upper limit of the 95% confidence interval for the median survival time could not be determined because the estimated upper confidence limit for the survival function for this group never falls below 0.5 [19].

**Figure 2:**
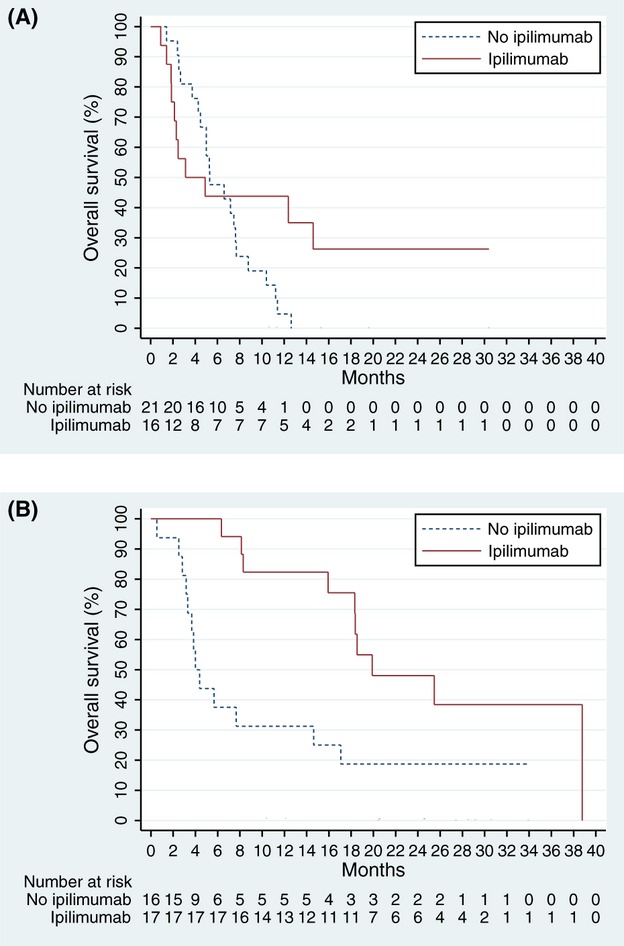
Censored overall survival of patients for each type of radiation therapy by ipilimumab treatment. (A) WBRT: treatment with ipilimumab was not associated with survival in the subset of patients who underwent WBRT (HR = 0.56, *P* = 0.15); (B) SRS: treatment with ipilimumab was significantly associated with improved survival in the subset of patients who underwent SRS (HR = 0.31, *P* = 0.009). WBRT, whole brain radiation therapy; HR, hazard ratio; SRS, stereotactic radiosurgery.

Exploratory subgroup analyses by type of RT revealed an apparent initial decrement in the survival of the WBRT patients who received ipilimumab until ∼6 months, at which point the survival curves cross (Fig. 2A). Treatment with ipilimumab was not significantly associated with survival in the WBRT subset (HR = 0.56, *P* = 0.15). The median survival of WBRT patients who did or did not receive ipilimumab was 3.1 versus 5.3 months, respectively (*P* = 0.60, not significant; Table 2). Among patients who underwent SRS, treatment with ipilimumab was significantly associated with improved survival (HR = 0.31, *P* = 0.009; Fig. 2B). These patients had a censored median survival of 19.9 months, whereas the censored median survival in patients who underwent SRS but did not receive ipilimumab was 4.0 months (Table 2), and this difference was statistically significant (*P* = 0.009). Among the SRS subset, we found no significant differences in the patient characteristics except for Eastern Cooperative Oncology Group (ECOG) performance status (*P* = 0.043), neurologic symptoms (*P* = 0.013), subsequent brain RT (*P* = 0.016), and BRAF inhibitor treatment (*P* = 0.049), all favoring the ipilimumab group. To explore the possibility that the combination of treatment with ipilimumab and SRS would improve the outcomes of interest, we tested the significance of the interaction between these two treatment indicators in the Cox regression model. Although the estimated hazard ratio for treatment with ipilimumab was 0.32 in the SRS treatment group as compared to 0.57 in the group that did not receive SRS, the interaction was not statistically significant (*P* = 0.31); however, there was insufficient power (19%) to detect a significant interaction in a post hoc power calculation.

Due to the fact that subsequent therapies may have impacted the OS, the effect of ipilimumab on response rate to brain RT and TTP_br_ was also analyzed. Forty-four patients were evaluable for response using irRC. Response rate and TTP_br_ using RECIST was similar, except for three patients (two in the ipilimumab groups and one in the comparison) who were classified as stable disease rather than progressive disease (not shown). There were no CRs (Table 3). The responses of patients who received ipilimumab versus the comparison groups were not significantly different using Fisher's exact test (*P* = 0.238; Table 4). Partial response to RT was observed in two of 22 patients (9.1%) in the comparison groups, both of whom were treated with WBRT. Among patients who were treated with the first dose of ipilimumab prior to RT, partial responses to RT were observed in four of 10 patients (40.0%), including two who were treated concurrently. This is in contrast to two of 12 responses (16.7%) among patients who received their first dose of ipilimumab after RT. Notably, one patient started ipilimumab soon after completing WBRT (within 3 weeks), and demonstrated an 80% reduction in her dominant brain metastasis and complete disappearance of four smaller ones. The distribution of responses of patients who received ipilimumab before versus after RT was not significantly different using Fisher's exact test (*P* = 0.224).

**Table 3:** Response to RT by type of RT

	WBRT	SRS
Complete response	0/27 (0%)	0/17 (0%)
Partial response	5/27 (18.5%)	3/17 (17.6%)
Stable disease	13/27 (48.1%)	7/17 (41.2%)
Progressive disease	9/27 (33.3%)	7/17 (41.2%)

RT, radiation therapy; WBRT, whole brain radiation therapy; SRS, stereotactic radiosurgery.

**Table 4:** Response to RT by ipilimumab treatment

	No ipilimumab	Ipilimumab before RT	Ipilimumab after RT
Complete response	0/22 (0%)	0/10 (0%)	0/12 (0%)
Partial response	2/22 (9.1%)	4/10 (40.0%)	2/12 (16.7%)
Stable disease	13/22 (59.1%)	2/10 (20.0%)	5/12 (41.7%)
Progressive disease	7/22 (31.8%)	4/10 (40.0%)	5/12 (41.7%)

RT, radiation therapy.

No unexpected toxicities from radiation were observed in these patients. Intratumoral hemorrhage within 30 days after the start of RT was observed in 4/32 (12.5%) patients in the comparison groups and 1/25 (3.9%) in the ipilimumab groups. Three instances of radiation necrosis were observed, which were all patients in the comparison groups.

## Discussion

The results of this study are consistent with other limited retrospective data demonstrating that ipilimumab and SRS are associated with significantly improved survival [8]. In a previous analysis of 77 patients treated with SRS, the reported median OS was 21.3 months for patients treated with SRS and ipilimumab, versus 4.9 months for patients who received SRS alone. In another retrospective study of ipilimumab and SRS, there was no statistically significant survival benefit at 6 months, but patients in that analysis did poorly, as demonstrated by a median OS of 5.9 months and a 30% rate of intracranial hemorrhage [9]. In the present study, the survival of the SRS and ipilimumab group was significantly longer than that for SRS alone (median of 19.9 months vs. 4.0 months; *P* = 0.009), with a HR of 0.31 that was statistically significant (*P* = 0.009). Neither SRS nor ipilimumab treatment individually appears to account for the prolonged survival seen in this analysis. These survival outcomes compare favorably with the reported survival for patients with MBM who received monotherapy with either ipilimumab or SRS, which range from 5 to 7 months [3, 4, 7]. As an adjunct comparison, we analyzed 20 serial melanoma patients (2011–2012) without brain metastases who received ipilimumab at the University of Michigan and found that their median survival was 15.5 months. These findings strongly support the combined use of both ipilimumab and radiotherapy for patients with MBM.

The magnitude of the 16-month survival benefit of both ipilimumab and SRS treatment over SRS without ipilimumab suggests that the two treatments may be synergistic (rather than having independent additive effects). We found a larger benefit of ipilimumab for those patients who received SRS, but we did not have adequate power to determine whether this interaction was statistically significant. The theory of synergy between ipilimumab and RT is supported by preclinical and clinical data. Although the central nervous system previously has been thought to be an immune sanctuary, it is now known that activated lymphocytes can cross through the blood–brain barrier [20]. Preclinical data demonstrate that ionizing radiation increases the permeability of the blood–brain barrier [21], induces the presentation of previously occult cancer antigens to T cells [22], and generates tumor-specific cytotoxic T lymphocytes [23, 24]. In patients with MBM, a high level of T-cell immune infiltrate in the tumors is associated with prolonged survival [25]. In mice, fractionated RT to a tumor on one flank with concurrent administration of anti-CTLA-4 antibody induced activated tumor-specific T cells and inhibited growth of tumors on the contralateral flank, located outside of the radiation field [26]. This phenomenon, the regression of tumors at sites distant to the irradiated site, is known as the abscopal effect. The abscopal effect was documented in a patient with metastatic melanoma who was treated with ipilimumab for 15 months before she required palliative radiation to a paraspinal mass [27]. She experienced regression of tumors in the hilar lymph nodes and spleen, accompanied by temporal increases in antibodies to cancer-testis antigen NY-ESO and activation of CD4+ T cells, suggesting that the both humoral and cell-mediated immunity play a role in the abscopal effect. Other cases have been reported, including a case of a man with brain metastases and nodal metastases who had a CR to concurrent treatment with ipilimumab and SRS and developed antibodies to the tumor antigens MAGEA3 and PASD1 [28].

The dose and schedule of RT may be important factors in spurring an immune response, but this is not well understood. In mouse models of breast cancer and colon cancer, growth inhibition of tumors outside the radiation field occurred only when anti-CTLA-4 antibody was given concurrently with fractionated radiation, but not single-dose radiation [26]. To the contrary, our analysis suggests that SRS is superior to fractionated WBRT; however, this may be confounded by the fact that the SRS patients had fewer brain metastases, and, in general, a better expected survival. Interestingly, in the mouse study, RT of three doses of 8 Gy was more effective at synergizing with the anti-CTLA-4 antibody than five doses of 6 Gy. Our analysis did not include any comparable oligofractionated regimens, as they are not used clinically for intact brain metastases very often.

Limitations of this study include small sample sizes, retrospective data collection, and selection bias, meaning that patients who had more indolent disease may have been more likely to receive ipilimumab. The comparison groups and the ipilimumab groups were extremely similar by all three prognostic indicators (Table 1), but we found that the subset that received SRS and ipilimumab had better performance status at baseline and were more likely to be neurologically asymptomatic, which may have impacted our findings. While our favorable survival results could be partially explained by selection bias, we attempted to include all serial cases of patients in our census who received ipilimumab, including those with leptomeningeal involvement and those who were rapidly progressing. Six patients received only one or two doses of ipilimumab, generally because they became too ill to receive the full course of treatment.

Due to the small sample size, we only included ipilimumab treatment and type of RT in our main Cox regression model [29]. Patient characteristics were examined for differences in factors that may have contributed to the survival outcomes (Table 1). Patients in the ipilimumab groups received subsequent brain RT significantly more frequently. To the best of our knowledge, salvage RT for MBM has not been shown to improve survival, and thus we did not include subsequent RT in the multivariate model. There was also significantly more exposure to BRAF inhibitor therapy in the ipilimumab groups. BRAF inhibition has a documented survival advantage in metastatic melanoma without brain metastases [30]. Dabrafenib appears to be active in MBM, and median survival was ∼8 months in patients with either untreated or progressive MBM despite local treatment [31]. Thus, we included BRAF-inhibitor exposure in the multivariate model, but the effect of ipilimumab overshadowed it (HR = 0.50 for ipilimumab treatment vs. HR = 0.95 for BRAF inhibitor treatment; *P* = 0.03 and *P* = 0.91, respectively). One explanation for the lack of significance for BRAF inhibitor treatment may be the strong correlation between treatment with BRAF inhibitors and treatment with ipilimumab. Therefore, even when considering the higher use of BRAF inhibitors and subsequent RT, the patients in our analysis had a survival that is arguably longer than expected.

We did not see evidence of increased toxicity with the combination treatment. Although intratumoral hemorrhage [14] and radiation necrosis [15] have been reported, we did not find that there was excess toxicity in patients who received ipilimumab. In fact, the rate of intratumoral hemorrhage was higher in the comparison group. There were five patients who were treated concurrently with ipilimumab and RT, one of whom had intratumoral hemorrhage. There was a slight initial decrement in survival after WBRT that was observed in patients who received ipilimumab. This does not appear to be explainable by increased toxicity but instead may be due to treatment of patients with significantly advanced disease with lower DS-GPA scores (mean DS-GPA 1.7 vs. 2.2, data not shown).

It is not clear what the ideal timing of ipilimumab with respect to RT is, as survival and response outcomes were conflicting in subgroup analyses of treatment sequences. Response rates were higher in the ipilimumab group, especially when ipilimumab was given prior to RT (40% vs. 17% in the ipilimumab after RT group and 9% in the comparison groups that did not receive ipilimumab). Among the six responding patients in the ipilimumab group, four of them were treated with ipilimumab prior to RT (including two treated concurrently), and the fifth received ipilimumab shortly after completing WBRT. In a subgroup analysis, survival seemed to be improved for the patients who received ipilimumab after RT as compared to patients who received ipilimumab prior to RT (median of 18.4 months vs. 8.1 months). OS is affected by multiple factors, and favorable selection bias likely applies more to the groups that received ipilimumab after RT, as some of the patients received RT in 2009–2010 and they lived long enough to reach the FDA approval of ipilimumab in 2011. The survival data and response data for sequence of ipilimumab and RT are somewhat conflicting, but the response data are likely more informative in this regard. Sequence will be important to explore in future clinical trial designs. Previous studies support the approach of immunotherapy prior to RT. In the preipilimumab era, immunotherapy prior to SRS was associated with statistically significant gains in survival in other retrospective studies [4, 32], but not all [3]. In 333 patients who underwent SRS for MBM, the history of prior immunotherapy (interleukin-2 or interferon), but not subsequent immunotherapy, was associated with improved survival of 13.8 months, as compared to 5.8 months in the group that did not receive prior immunotherapy [4]. Assuming that OS in the ipilimumab after RT group is more susceptible to selection bias, and taking into account the available positive data on immunotherapy prior to SRS, our data lend weight to the idea that pretreatment with ipilimumab before RT (and perhaps given shortly after RT) may improve the rate of response to RT; the impact on survival is unclear but deserves further study.

## Conclusions

In our analysis, ipilimumab therapy was associated with improved OS in patients with MBM who received RT, and the median survival of patients in the ipilimumab and SRS group was nearly five times the group who received SRS alone. This is the second single-institution retrospective study to report a 16-month survival benefit for this population [8]. The magnitude of the benefit suggests that the effect of ipilimumab could be synergistic with focused high-dose RT. The optimal sequence of combination therapy with ipilimumab and RT is not known, but a multimodality approach appears to be essential to optimize patient outcomes. Prospective studies are on-going.
